# The interleukin‐1α stimulated expression of the wrinkle‐inducing elastase neprilysin in adult human dermal fibroblasts is mediated via the intracellular signaling axis of ERK/JNK/c‐Jun/c‐Fos/AP‐1

**DOI:** 10.1111/1346-8138.17520

**Published:** 2024-10-31

**Authors:** Mariko Takada, Uma Chandula Pinnawala, Shinichi Hirano, Genji Imokawa

**Affiliations:** ^1^ Center for Bioscience Research and Education Utsunomiya University Utsunomiya Tochigi Japan; ^2^ TourtVert Co, Ltd Minoh Osaka Japan

**Keywords:** human fibroblasts, interleukin‐1α, intracellular signaling, neprilysin, wrinkles

## Abstract

Neprilysin is a skin wrinkle‐inducing membrane bound elastase that is expressed abundantly in UV‐exposed and in aged dermal fibroblasts. The overexpression of neprilysin is closely associated with enhanced epithelial‐mesenchymal cytokine interactions mainly via interleukin (IL)‐1α, which has the distinct potential to stimulate the expression of neprilysin by human dermal fibroblasts (HDFs). The over‐expression of neprilysin also accelerates the formation of wrinkles, accompanied by disruptions of the three‐dimensional architecture of dermal elastic fibers that are responsible for the loss of skin elasticity. Because the signaling pathway(s) that lead to the IL‐1α‐stimulated expression of neprilysin in HDFs remain unclear, we characterized the signaling pathway involved, including their related transcription factors, in IL‐1α‐treated HDFs. Since qRT‐PCR analysis revealed that the mRNA expression level of neprilysin is stimulated to a stronger extent in adult HDFs (aHDFs) by IL‐1α than in neonatal HDFs, we used aHDFs for the signaling analysis. Western blotting analysis of the phosphorylation of signaling factors revealed that IL‐1α significantly stimulated the phosphorylation of ERK1/2, RSK, JNK, p38, MSK1, NFkB, c‐Jun, ATF‐2, CREB, and STAT3. Analysis using various signaling inhibitors demonstrated that inhibiting ERK and JNK but not p38, MSK1, NFkB, or STAT3 significantly abrogated the IL‐1α stimulated expression of neprilysin at the mRNA, protein, and enzyme activity levels. Furthermore, silencing c‐Fos significantly down‐regulated the IL‐1α‐increased expression of neprilysin at the protein and enzyme activity levels. These findings strongly suggest that the IL‐1α‐stimulated expression of neprilysin in aHDFs is mediated via the intracellular signaling axis of ERK/JNK/c‐Jun/c‐Fos/AP‐1.

## 1 INTRODUCTION

The most prominent symptom by which skin aging is distinctly recognized is wrinkles and sagging of facial skin. Our clinical evaluation of human skin revealed that the severity of wrinkles in the corner of the eye is distinctly associated with a deficiency in skin elasticity at the same corner of the eye,^1^, ^2^, ^3^ which strongly suggested that the formation of facial wrinkles and sagging is mechanistically attributed to the loss of elastic properties of the skin. Correspondingly, our animal studies^3^, ^4^, ^5^ demonstrated that repetitive exposure of the skin to ultraviolet (UV)B at sub‐erythemal doses continues to impair its elastic properties, which subsequently results in the formation of wrinkles at a certain threshold level of decreased elasticity. The sum of our clinical human and animal studies strongly suggested that facial wrinkles and sagging are promoted by the preceding deficiency of skin elasticity which is provoked by repeated sunlight exposure. To elucidate the possible functions of elastic fibers responsible for skin elasticity, we used scanning electron microscopy to observe the three‐dimensional configuration of elastic fibers and to determine the effects of repeated UVB irradiation on the three‐dimensional configuration of elastic fibers in rat hindlimb skin.^3^, ^6^, ^7^ These results suggested that the deformations in elastic fiber configuration are mechanistically associated with the marked decline in skin elasticity in situ because the tight fit of rat skin is based on the ability of elastic fibers to resume their tight configuration after being stretched.

The most plausible mode of action for the alteration of elastic fiber networks was that repetitive UVB exposure at sub‐erythemal doses enhances the activity of elastase in the dermis via unknown mechanisms, which impairs the elastic fibers by which fibroblasts are anchored via their plasma membrane to the surrounding dermal connective matrix tissue. To determine the epistatic connection between the stimulated activities of elastase and the down‐regulated skin elastic properties as well as the deformation of the three‐dimensional configuration of elastic fiber networks, we investigated the effects of ovariectomy on accelerated aging phenomena such as skin wrinkling and on the UVB sensitivity that leads to the increased wrinkle formation.^3^, ^5^ Based on the above evidence, we concluded that the deformation of the three‐dimensional configuration of the elastic fiber network can be mainly ascribed to a significant increase in elastase activity but not in the activities of collagenases I or IV in the dermis of ovariectomized mice compared with sham‐operated control mice.^3^, ^5^

To clarify the mechanistic relationship between the up‐regulated elastase activity and wrinkle formation, we designed a new specific elastase inhibitor, N‐phenethylphosphonyl‐L‐leucyl‐L‐tryptophane (NPLT), which has no inhibitory effect on other matrix degrading proteinases, such as type I collagenase, type IV collagenase or neutrophil elastase.^8^ Therefore, to elucidate the essential role of the up‐regulated levels of skin fibroblast‐derived elastase in the impairment of the three‐dimensional architecture of the elastic fiber network required to maintain a normal level of skin elasticity, we performed an in vivo inhibition animal study using NPLT.^3^, ^8^ We determined the effects of NPLT on wrinkle formation, the loss of skin elasticity and the degeneration in the three‐dimensional configuration of elastic fibers as well as their coordinated mutual linkages.^9^, ^10^ While repeated UVB irradiation enhances the activity of fibroblast‐derived elastases in the dermis, topical post‐application of the synthetic inhibitor NPLT that is specific for fibroblast elastase on the UVB‐exposed skin completely prevented wrinkle formation,^11^ accompanied by an abrogating effect on the deficiency of skin elastic properties.^3^, ^8^ As for the effects of different concentrations of NPLT, there was a close and significant interrelationship among wrinkle formation, skin elasticity, and elastic fiber linearity.^9^, ^10^ Those in vivo NPLT inhibition studies convincingly demonstrated that the enhanced elastase activity of dermal fibroblasts plays a pivotal role in the degeneration of elastic fibers in chronically UVB‐exposed dermis and that the loss of skin elasticity occurs as a direct result of the altered elastic fiber configuration, which subsequently triggers the formation of skin wrinkles and sagging.

The inhibitory effect of NPLT on wrinkle formation was corroborated by human clinical studies^3^, ^10^ using an extract of *Zingiber officinale* (L.) Rose, which is capable of inhibiting skin fibroblast‐derived elastase but does not inhibit neutrophil elastase. After 1 year of topical application of that extract, the wrinkle score and ratio (%) of the wrinkle area assessed using image analysis were significantly ameliorated at the corners of the eyes on the side of the face treated with the extract compared to those on the placebo‐treated side, accompanied by a significant increase in skin elasticity (Ur/Uf).^3^, ^10^ These clinical results suggested that the extract of *Zingiber officinale* (L.) Rose abrogated the decreased skin elasticity and diminished wrinkle formation by inhibiting the fibroblast‐derived elastase activity.

While the above findings strongly suggested that skin fibroblast‐derived elastase plays a critical role in the formation of wrinkles through the impairment of the three‐dimensional architecture of the elastic fiber network, there was some evidence that skin fibroblasts synthesize an “elastase,” but its specific enzyme species and enzymatic and molecular properties were unknown. Fortunately, we noticed that there were several similarities between skin fibroblast‐derived elastase and neprilysin in terms of their size (97 000 Da), their membrane‐bound nature and their inhibitory profiles.^12^ We then demonstrated that the skin fibroblast‐derived elastase responsible for wrinkles and sagging formation is identical to membrane‐bound metalloproteinase, neprilysin.^13^ The elastin‐degrading property of neprilysin has been corroborated by other studies,^14^, ^15^ the latter of which used mass spectrometry and bioinformatics approaches to demonstrate that elastin fibers from older donors could be cleaved more efficiently than those from young donors or intact skin elastin.

That identification allowed us to characterize the epithelial‐mesenchymal paracrine cytokine interactions between UVB‐exposed‐epidermal keratinocytes and dermal fibroblasts, by which the expression of neprilysin is highly stimulated in dermal fibroblasts. Therefore, using co‐cultures of cell plates seeded with human dermal fibroblasts (HDFs) and cell inserts seeded with human keratinocytes, we found that interleukin‐1α (IL‐1α) and granulocyte macrophage colony stimulatory factor (GM‐CSF) are intrinsic cytokines that are secreted by UVB‐exposed keratinocytes and penetrate into the dermis and subsequently stimulate the expression of neprilysin by HDFs.^12^, ^16^ The sum of those effects would clearly increase the formation of wrinkles in the skin but the mechanism(s) involved needed to be clarified.

Based on those newly discovered biological mechanisms underlying the UVB‐induced wrinkle formation, our final goal was to characterize the intracellular signaling mechanisms involved in the up‐regulated expression of neprilysin by HDFs, which would lead to developing effective candidate materials that could act as anti‐wrinkle agents. Since IL‐1α has a higher potential to stimulate neprilysin expression by HDFs than does GM‐CSF,^13^ in this study, we determined the signaling mechanisms involved in the IL‐1α‐stimulated expression of neprilysin by HDFs. Our study demonstrates for the first time that the up‐regulated expression of neprilysin in IL‐1α‐treated HDFs is mediated via the ERK/JNK/c‐Jun/c‐Fos/AP‐1 signaling axis.

## 2 MATERIALS AND METHODS

### 2.1 Materials

The primary antibodies phospho‐SAPK/JNK (no. 9251), SAPK/JNK (no. 9252), phospho‐p38 MAPK (Thr180/Tyr182) (no. 9215S), p38 MAPK (no. 9212S), phospho‐CREB (Ser 133) (no. 9198S), phospho‐STAT3 (Ser 727) (no. 9134S), phospho‐NFkB p65 (Ser536) (no. 3033S), NFkB (no. 8242), phospho‐c‐Jun (no. 3270S), c‐Jun (no. 9165S), phospho‐p44/42 MAPK (Erk1/2) (Thr202/Tyr204) (no. 9101), p44/42 MAPK (Erk1/2) (no. 9102), phospho‐ATF‐2 (no. 9221S), ATF‐2 (no. 9226S), and c‐Fos (no. 2250S) were purchased from Cell Signaling Technologies (MA, USA). Phospho‐MSK1 (no. 32190) was obtained from Abcam (Cambridge, UK). The CD10 (F‐4) antibody (NEP) (no. sc‐46 656) and SB239063, an inhibitor of p38, (no. SC220094) was purchased from Santa Cruz Biotechnology (TX, USA). Anti‐rabbit IgG (no. NA934V) and anti‐mouse IgG (no. NA931V) were obtained from GE Healthcare (IL, USA). IL‐1α, human recombinant animal‐derived free (no. 098‐06801) was purchased from Fujifilm Wako Pure Chemicals LTD (Osaka, Japan). β‐Actin (no. A5316) and JNK inhibitor ii (no. 420119) were from Sigma‐Aldrich (MO, USA). The MEK inhibitor (U0126) (no. V112A) was from Promega (WI, USA) and NFkB activation inhibitor II (JSH‐23) was from Calbiochem (CA, USA).

### 2.2 Cell culture

Adult normal human primary dermal fibroblasts (aHDFs) (ATCC, VA, USA) were cultured in Fibroblast Basal Medium with Fibroblast Growth kit low serum (ATCC, VA, USA) at 37°C in a 95% air, 5% CO_2_ atmosphere. HDFs derived from human foreskins (nHDFs) (Thermo Fisher Scientific, Waltham, MA, USA) were cultivated in Dulbecco's modified Eagle's medium (DMEM) with 10% fetal bovine serum (FBS) at 37°C in a 95% air, 5% CO_2_ atmosphere.

### 2.3 Neprilysin activity assay

Neprilysin activity was determined using a Fluorimetric SensoLyte 520 kit (AnaSpec, CA, USA). The neprilysin substrate (1 mM component A) was dissolved in 1 × assay buffer, then was mixed with 1 × assay buffer with the sample in 96 well plates. Fluorescence was measured using microplate reader (Tecan Spark 10 M; Männedorf, Switzerland) at an excitation wavelength of 485 nm, and an emission wavelength of 535 nm. Neprilysin activity was expressed as relative fluorescent unit (RFU).

### 2.4 Western blotting

aHDFs were cultured (1.5 × 10^5^ cells per mL) for the indicated times and then were extracted using RIPA buffer (Fujifilm Wako Pure Chemicals Ltd.) plus halt protease and phosphatase inhibitor cocktail (Thermo Fisher Scientific). Extracted total proteins were separated by western blotting. Samples were separated on SDS polyacrylamide gels, transferred onto immune‐blot polyvinylidene fluoride (PVDF) membranes (Bio Rad, CA, USA), blocked with blocking one (Nacalai tesque, Kyoto, Japan) and probed with primary antibodies dissolved in Can get solution1 (Toyobo no. NKB‐101, Osaka, Japan) at 4°C overnight; β‐actin was used as the loading control. The membranes were washed with tris‐buffered saline with Tween (TBST) and probed with either anti‐rabbit IgG or anti‐mouse IgG dissolved in Can get solution 2 (Toyobo) and developed by enhanced chemiluminescence (ECL) (GE Healthcare, IL, USA). Detection was performed using a Lumino Graph III WSE‐6300 (ATTO Corp., Tokyo, Japan). Protein expression was quantified from images obtained using a CSAnalyzer4 (ATTO Corp. Tokyo, Japan).

### 2.5 Real‐time qRT‐PCR

After treatment with IL‐1α for 24 h, neprilysin mRNA levels were measured using real‐time qRT‐PCR. Total RNAs were isolated using a ReliaPrep™ RNA Miniprep System (Promega, WI, USA) followed by reverse transcription to cDNA by ReverTra Ace® qPCR RT Master Mix (Toyobo). Real time PCR was performed using SYBR Fast qPCR Mix (Takara Bio, Shiga, Japan). PCR amplification was performed using a LightCycler96 (Roche Diagnostic, Basel‐Stadt, Switzerland). The primers used are shown in Table 1. Relative gene expression levels were normalized to Ribosomal Protein Lateral Stalk Subunit P0 (RPLP0) expression levels.

**TABLE 1:** Forward and reverse (5′ → 3′) sequences of the primers used in the study.

Primer	Sequence	
RPLP0	Forward	5′‐TTCGACAATGGCAGCATCTACAA‐3′
RPLP0	Reverse	5′‐CTGCAGACAGACACTGGCAACA‐3′
Neprilysin	Forward	5′‐GGGAGCTGATGAAACTGACAAATG‐3′
Neprilysin	Reverse	5′‐TCTCTGGACAGCTTGCACCTAC‐3′

### 2.6 siRNA transfection

aHDFs were seeded in 12‐well plates (6 × 10^4^ cells per well) and were cultured for 1 day. The next day, the aHDFs were transfected with control siRNA (MISSION® siRNA Universal Negative Control, Sigma‐Aldrich, MO, USA) or siRNA c‐Fos (Sigma‐Aldrich, MO, USA) using Lipofectamine® RNAiMAX (Invitrogen™, CA, USA) for 2 days following the manufacturer's protocol.

### 2.7 Statistics

All experiments were repeated independently at least three or more times. Results are expressed as means + standard deviation as indicated in the figure legends. Student's *t*‐test was used for two pair comparisons. Tukey's multiple comparison test was used for multiple comparisons. Differences at *P* < 0.05 are considered statistically significant.

## 3 RESULTS

### 3.1 HDF cell lines and their sensitivity to cytokines

**FIGURE 1:**
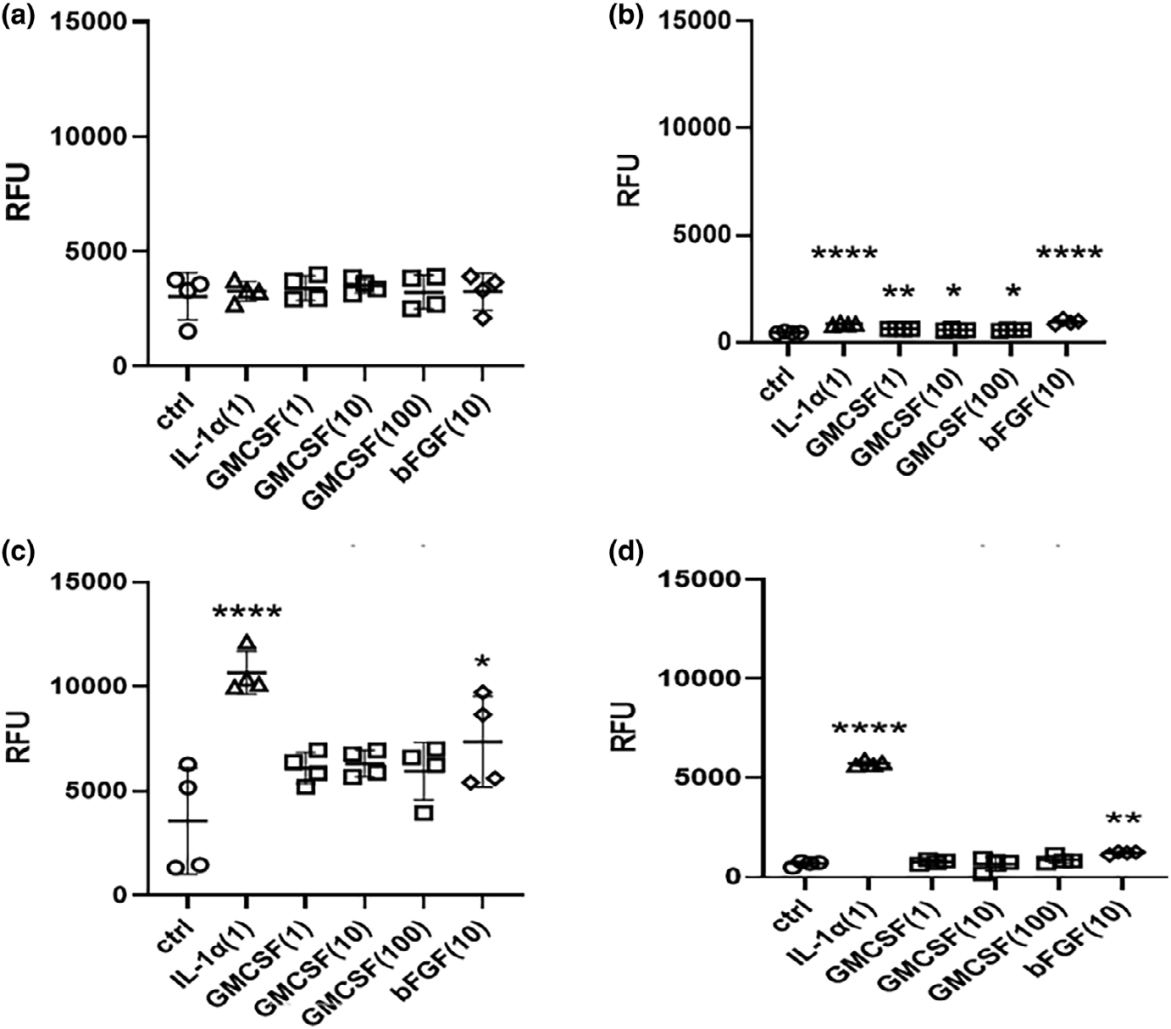
Effect of IL‐1α, GM‐CSF, and bFGF on neprilysin activity (RFU) in nHDFs and aHDFs. (a) NHDFs 24 h, (b) AHDFs 24 h, (c) NHDFs 72 h, (d) AHDFs 72 h nHDFs, and aHDFs were incubated with IL‐1α, GM‐CSF or bFGF for 24 or 72 h after which the cell lysates were subjected to neprilysin activity assay. (*x*) indicates concentrations of ng/mL. Neprilysin activity is expressed as RFU as described in the Section 2. Data represent means ± standard deviation, *n* = 4; ****P < 0.0001, **P < 0.01, *P < 0.05 by Tukey's multiple comparisons test.

Neonatal HDFs (nHDFs) and adult HDFs (aHDFs) were compared for the stimulatory effects of IL‐1α, GM‐CSF, and bFGF on neprilysin activity. Whereas treatment of nHDFs for 24 h with IL‐1α, GM‐CSF or bFGF did not cause any significant increase of neprilysin activity, treatment of nHDFs for 72 h elicited a significant increase by IL‐1α and by bFGF (Figure 1a,c). On the other hand, while treatment of aHDFs for 24 h with IL‐1α, GM‐CSF or bFGF caused slight but significant increases of neprilysin activity, treatment of aHDFs for 72 h elicited a slight increase by bFGF and a dramatic (~10‐fold) increase by IL‐1α (Figure 1b,d). During these comparative studies, we found that treatment of aHDFs with IL‐1α (1 ng/mL) for 72 h elicited the highest significant potential to stimulate neprilysin activity in nHDFs and aHDFs at both treatment times (Figure 1d), therefore aHDFs and IL‐1α as a stimulator were selected for further analysis.

### 3.2 Effects of IL‐1α on the activation of intracellular signaling cascades

**FIGURE 2:**
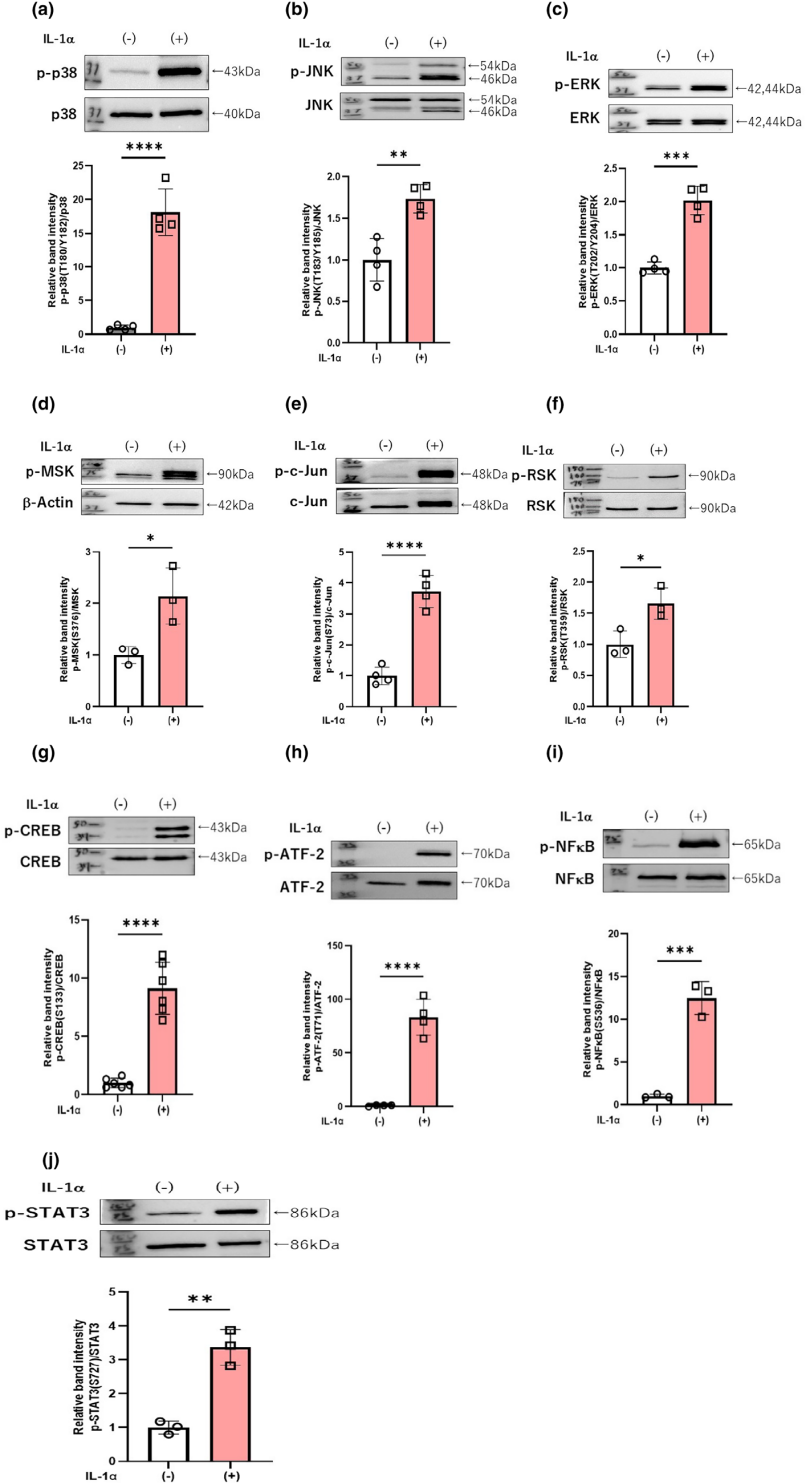
Effects of IL‐1α on the intracellular signaling cascades of p38 (a), JNK (b), ERK (c), MSK1 (d), c‐Jun (e), RSK (f), CREB (g), ATF‐2 (h), NFκB (i), and STAT3 (j) in aHDFs. aHDFs were incubated with or without IL‐1α (5 ng/mL) for 15 min and were then immunoblotted with antibodies to phosphorylated or non‐phosphorylated protein. Data represent means ± standard deviation, *n* = 3–4; ****P < 0.0001, ***P < 0.001, **P < 0.01, *P < 0.05 by *t*‐test.

Since there was no available published data for the stimulatory effects of IL‐1α on the phosphorylation of signaling factors in aHDFs, we first determined what intracellular signaling cascades are activated by IL‐1α in aHDFs. Western blot analysis of the phosphorylation of various signaling factors revealed that treatment with IL‐1α at 5 ng/mL significantly increased the phosphorylation of p38/ERK/RSK/JNK/ATF2/c‐Jun/MSK1/CREB/NFkB/STAT3 in aHDFs at 15 min post‐stimulation (Figure 2).

### 3.3 Effects of signaling inhibitors on the IL‐1α stimulation of neprilysin activity

**FIGURE 3:**
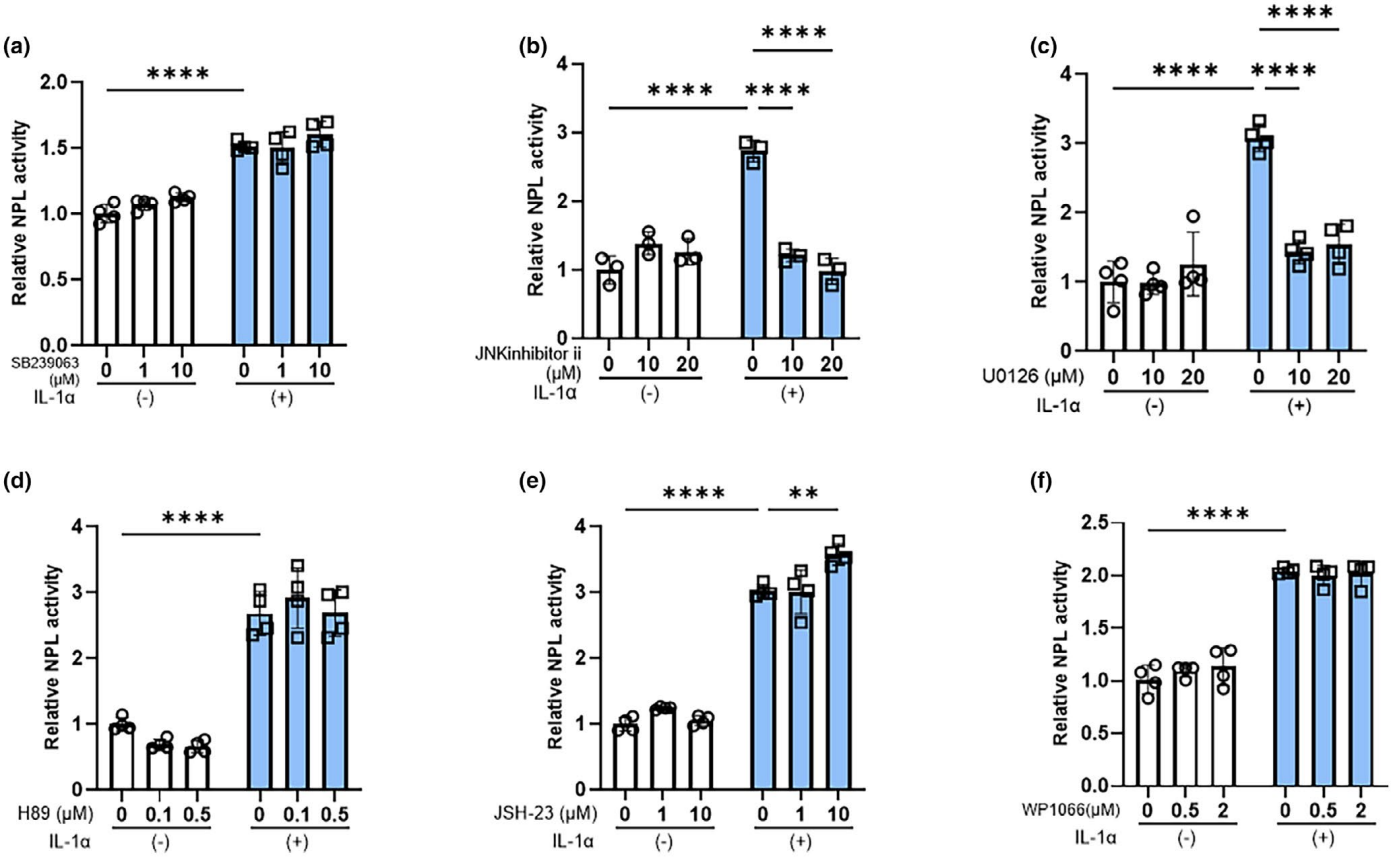
Effects of signaling inhibitors of p38 (a), JNK (b), MEK (c), MSK1 (d), NFκB (e), and STAT3 (f) on the IL‐1α stimulation of neprilysin activity. aHDFs were incubated with various signaling inhibitors with or without IL‐1α (5 ng/mL) for 72 h after which cell lysates were subjected to neprilysin activity assays. Data represent means ± standard deviation, *n* = 3–4; ****P < 0.0001, **P < 0.01 by Tukey's multiple comparisons test.

To determine the activated intracellular signaling cascades that are involved in the IL‐1α stimulation of neprilysin activity, we explored the inhibitory effects of various signaling inhibitors on the stimulated phosphorylation of IL‐1α‐activated signaling factors. These inhibition studies at the enzymatic activity level demonstrated that inhibitors of MEK (U0126) and JNK (inhibitor II) significantly abolished the IL‐1α‐stimulated neprilysin activity, whereas inhibitors of NFκB (JSH23), p38 (SB239063), MSK1 (H89), and STAT3 (WP1066) did not (Figure 3).

### 3.4 Effects of signaling inhibitors on IL‐1α‐stimulated mRNA levels of neprilysin

**FIGURE 4:**
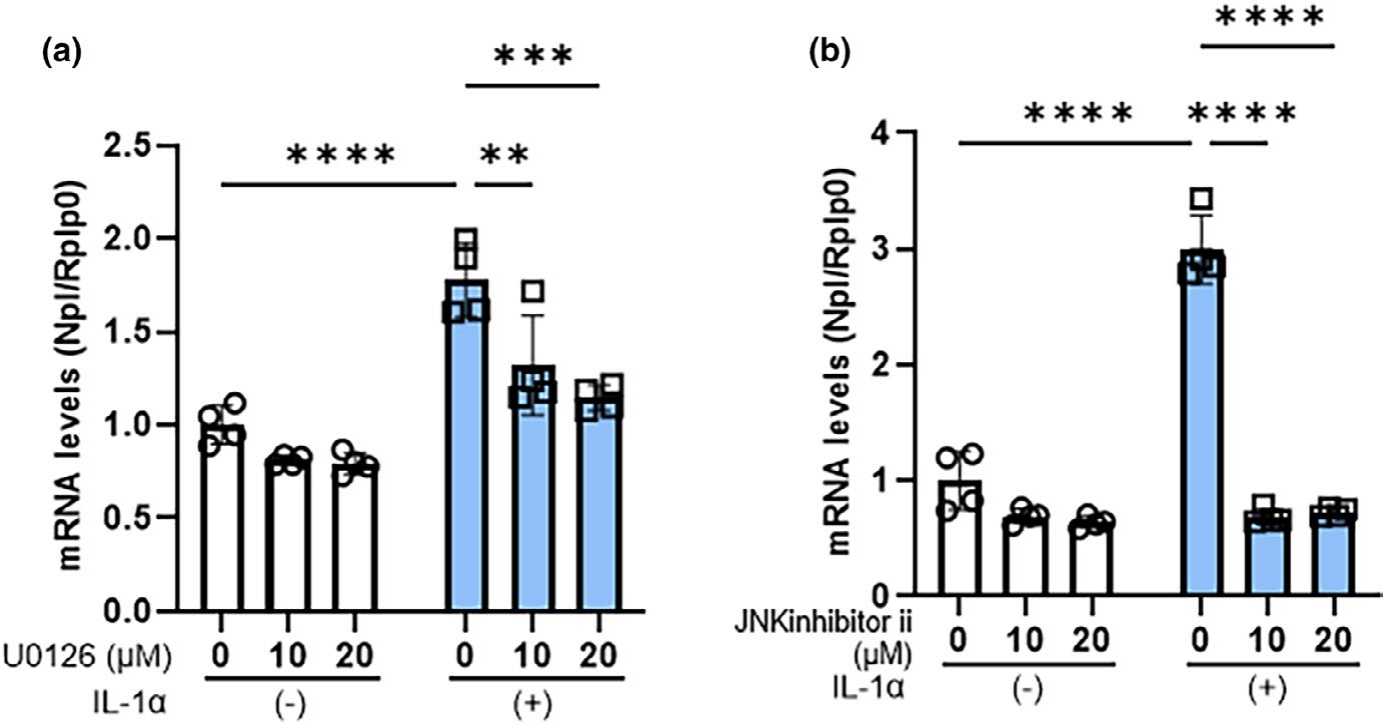
Effects of MEK (a) and JNK (b) inhibitors on the IL‐1α stimulated expression of neprilysin at the mRNA level. aHDFs were incubated with signaling inhibitors, with or without IL‐1α (5 ng/mL) for 24 h, after which mRNAs were extracted and converted to cDNAs followed by real time RT‐PCR analysis. Data represent means ± standard deviation, *n* = 4; ****P < 0.0001, ***P < 0.001, **P < 0.01 by Tukey's multiple comparisons test.

To corroborate the above inhibitory effects observed at the neprilysin enzymatic activity level, we next explored the effects of signaling inhibitors of MEK and JNK on the IL‐1α‐stimulated mRNA levels of neprilysin. Those inhibition studies at the mRNA level demonstrated that inhibitors of MEK and of JNK significantly abrogated the IL‐1α stimulated mRNA levels of neprilysin at 10 and 20 μM (Figure 4).

### 3.5 Effects of inhibitors of MEK and JNK on IL‐1α‐stimulated neprilysin protein expression

**FIGURE 5:**
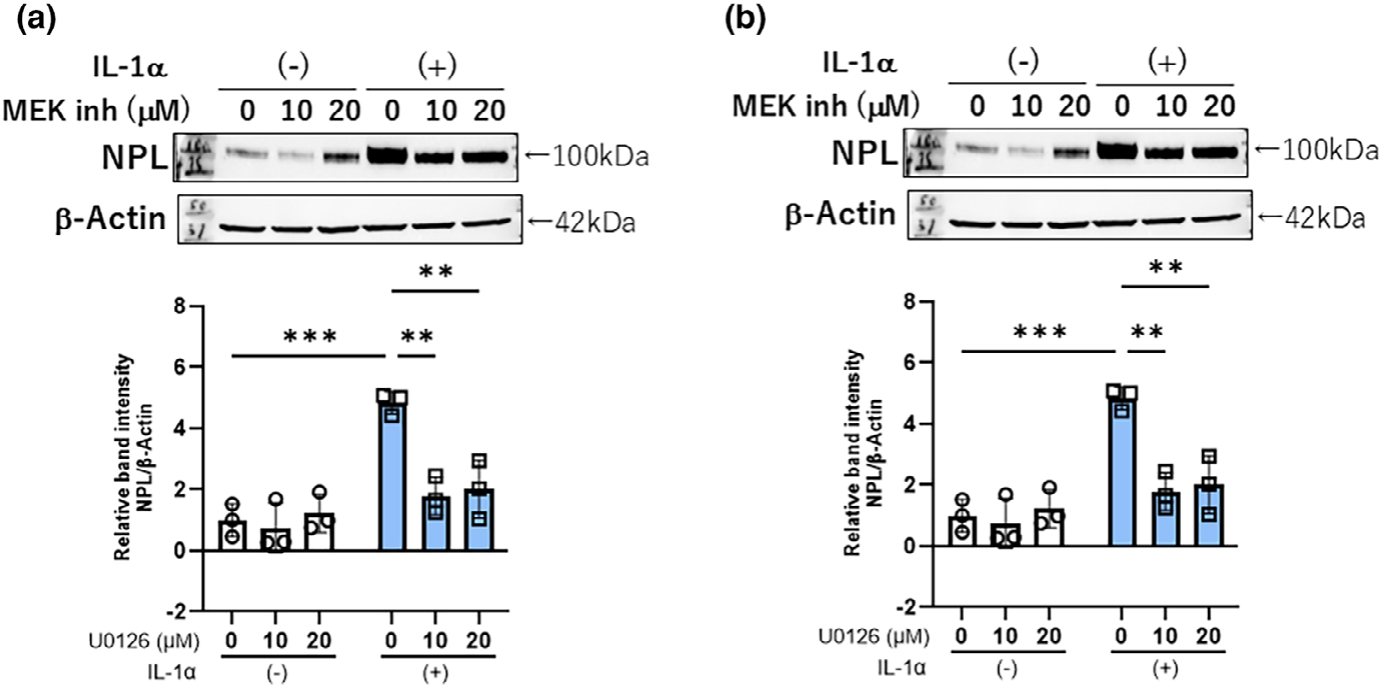
Effects of MEK (a) and JNK (b) inhibitors on the IL‐1α stimulated neprilysin protein expression. aHDFs were treated with signaling inhibitors with or without IL‐1α (5 ng/mL) for 72 h after which they were immunoblotted with a primary antibody to neprilysin; β‐Actin was used as a loading control. Data represent means ± standard deviation, *n* = 3; ****P < 0.0001, ***P < 0.001, **P < 0.01, by Tukey's multiple comparisons test.

We next explored the effects of inhibitors of MEK and JNK on IL‐1α‐stimulated neprilysin expression at the protein level. Western blotting revealed that MEK and JNK inhibitors significantly abrogated the IL‐1α stimulated neprilysin protein expression, which is consistent with our previous results for their effects on neprilysin activity as well as gene expression (Figure 5). Therefore, the precise analysis of signaling cascades and inhibition studies indicate the possible involvement of the intracellular signaling axis of ERK/JNK/c‐Jun/c‐Fos/AP‐1.

### 3.6 Effects of c‐FOS silencing on the IL‐1α stimulated neprilysin activity and protein expression

**FIGURE 6:**
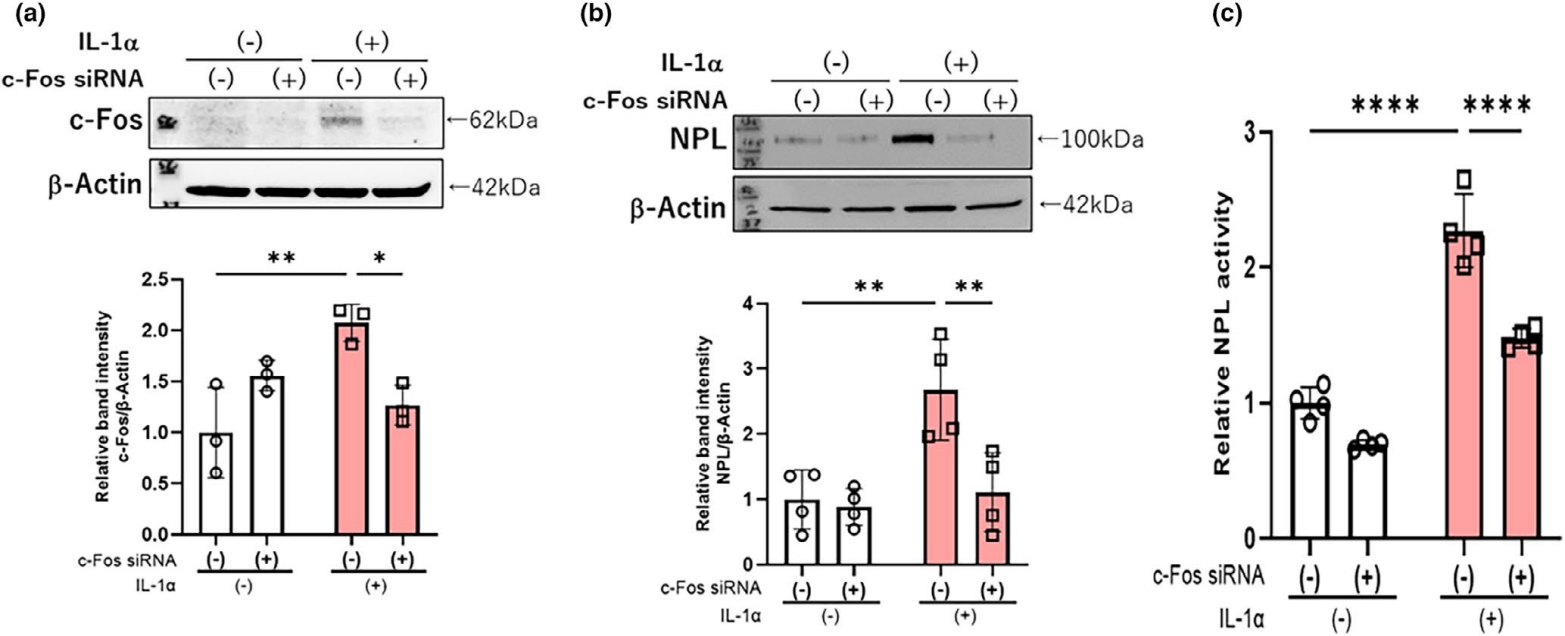
Effects of c‐FOS siRNA transfection. (a) Effects of c‐FOS siRNA transfection. After transfection with c‐FOS siRNA for 48 h, aHDFs were incubated with or without IL‐1α (5 ng/mL) for 2 h and were then immunoblotted with antibodies specific for c‐FOS and β‐Actin. (b) Effects of c‐FOS silencing on IL‐1α stimulated neprilysin protein expression. aHDFs were incubated with signaling inhibitors, with or without IL‐1α (5 ng/mL) for 72 h and were then immunoblotted with the primary antibody to neprilysin; β‐Actin was used as a loading control. (c) Effects of c‐FOS silencing on IL‐1α stimulated neprilysin activity. After transfection with c‐FOS siRNA for 48 h, aHDFs were incubated with or without IL‐1α (5 ng/mL) for 72 h and cell lysates were subjected to neprilysin activity assay. Data represent means ± standard deviation, *n* = 3–4; ****P < 0.0001, **P < 0.01, *P < 0.05 by Tukey's multiple comparisons test.

Since the above inhibition studies using signaling inhibitors indicated that the activated signaling cascade involved in the Il‐1α‐stimulation of neprilysin expression is mainly associated with the JNK/ERK/c‐Jun/cFos/AP‐1 axis, we next determined if c‐FOS silencing would abrogate the IL‐1α‐stimulation of neprilysin expression at the protein and enzymatic activity level. We found that c‐FOS siRNA transfection significantly abrogated the IL‐1α‐stimulated expression of neprilysin at the protein (Figure 6c) and enzymatic activity level (Figure 6b), accompanied by a significant abolishment of the IL‐1α‐enhanced c‐FOS protein (Figure 6a).

## 4 DISCUSSION

Based on the reported mechanism^13^, ^16^ by which skin wrinkle formation induced by UVB irradiation is mediated via the stimulated expression of neprilysin in dermal fibroblasts by cytokines including IL‐1α and GM‐CSF, which are released from UVB‐exposed keratinocytes and penetrate into the dermis, it was of considerable interest to determine which cytokine(s) are mainly responsible for stimulating the expression of neprilysin in HDFs. Further, it was important to know whether aHDFs are preferable to nHDFs in experiments to identify the intracellular signaling pathway(s) associated with the stimulated expression of neprilysin. In this approach, we found that although nHDFs have a higher activity of neprilysin at the baseline level than aHDFs, aHDFs are much more sensitive to cytokine stimulation than nHDFs, with IL‐1α being a more potent stimulator of neprilysin expression compared to GM‐CSF and bFGF. This finding prompted us to use IL‐1α and aHDFs in our signaling experiments, although the mechanisms involved in the difference of IL‐1α sensitivity between aHDFs and nHDFs remain unclear.

Since there is limited information describing the IL‐1α‐activated signaling pathway in aHDFs compared to nHDFs, we first determined the effects of IL‐1α on the phosphorylation of various signaling factors in aHDFs. We found that IL‐1α significantly up‐regulates the phosphorylation of the front line of the MAPKK axis, including JNK, ERK and p38 at 15 min post‐treatment, followed by the down‐stream axis of p38, MSK1/ CREB/ NFkB and ATF2, by the down‐stream axis of JNK and c‐Jun, and by the down‐stream axis of ERK, RSK and STAT3. Those IL‐1α‐activated signaling factors in aHDFs are very similar to those in UVB‐exposed human keratinocytes^17^, ^18^ in which the UVB‐released IL‐1α mainly triggers the activation of signaling factors. Of considerable interest is the fact that these activated signaling factors are also similar to factors stimulated by mycosporine‐like amino acids (MAAs), which were found to act as a stimulator for the mRNA expression of enzymes involved in the synthesis of the major dermal components, hyaluronan (HA), collagen, and elastin and also for HA secretion by nHDFs.^19^ In that study, MAAs were found to stimulate HA secretion by up‐regulating mRNA levels of HA synthase 2 through activation of an intracellular signaling cascade consisting of p38/MSK1/CREB/c‐Fos/AP‐1. On the other hand, the HA degrading enzyme‐associated protein HYBID was found to be up‐regulated by UVA irradiation at the mRNA and protein levels in nHDFs via the intracellular signaling pathway of the p38/ATF2 axis, which results in decreased HA secretion.^20^ MMP‐1 is well known to be a secretory type of metalloproteinase in fibroblasts, which can degrade collagen‐1 to play a role in photo‐aging.^21^, ^22^ The UV‐stimulated expression of MMP‐1 in fibroblasts is mediated via activation of the MAPK/c‐Jun/cFos/AP‐1 axis.^23^, ^24^, ^25^, ^26^

**FIGURE 7:**
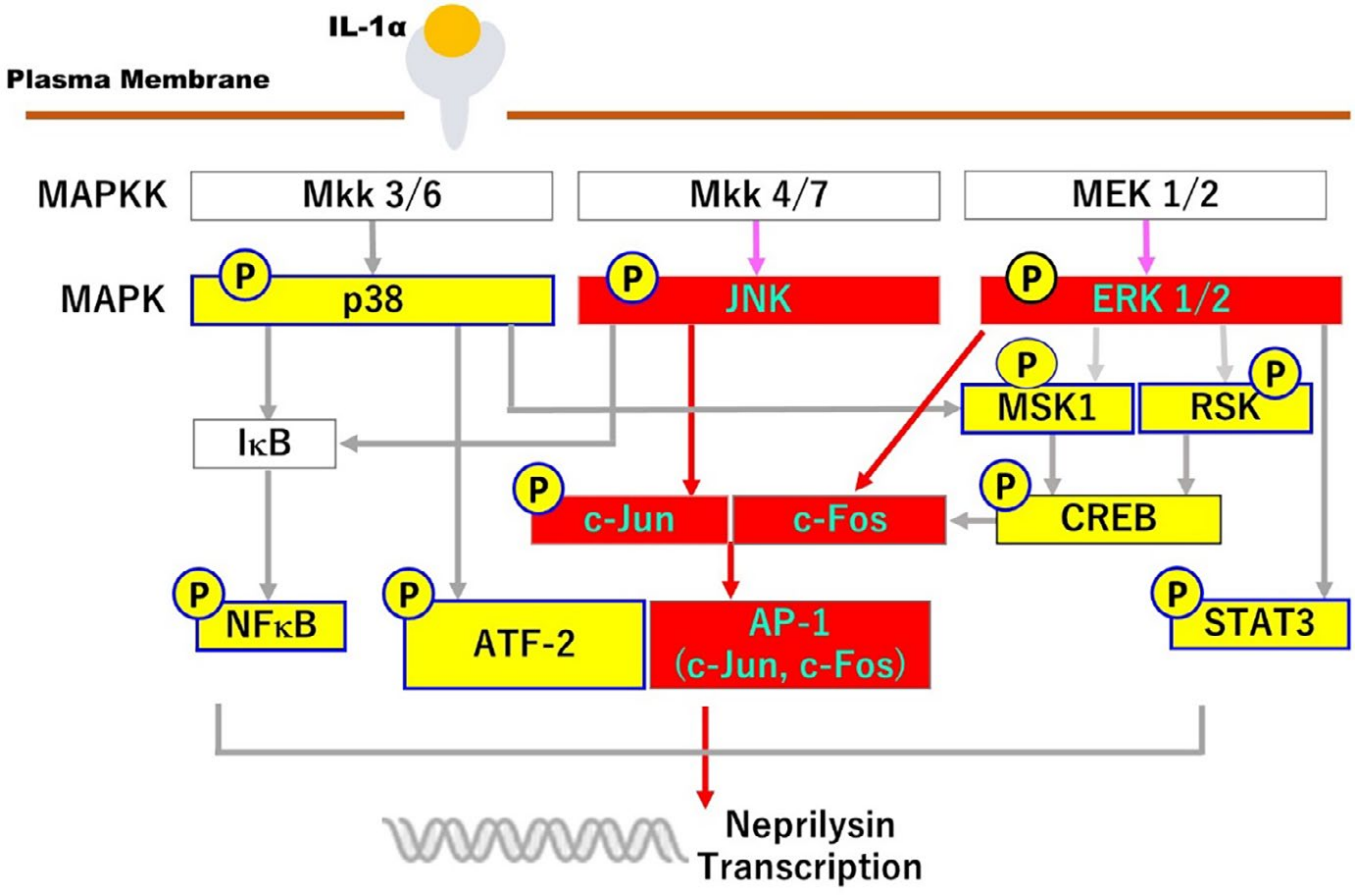
IL‐1α‐activated signaling cascades leading to the increased expression of neprilysin. Red rectangular box and red arrows indicate IL‐1α‐activated signaling cascades leading to the increased expression of neprilysin. “P” within a yellow circle represents the increased phosphorylation of each signaling factor by IL‐1α.

In research to identify the activated signaling pathway that leads to the IL‐1α‐stimulated expression of neprilysin, analysis using specific signaling inhibitors demonstrated that inhibitors of MEK and JNK significantly abrogate the IL‐1α‐increased activity of neprilysin, whereas inhibitors of p38, NFkB, MSK1, and STAT3 do not. Because H89 used as a MSK1 inhibitor can also inhibit RSK,^27^ our study also implied that the signaling axis of ERK/RSK/CREB is not involved in the IL‐1α‐stimulated expression of neprilysin. The abrogation of the stimulated neprilysin activity by MEK and JNK inhibitors was accompanied by the significant abolishment of increased mRNA and protein levels of neprilysin. Those signal inhibitory profiles strongly suggest that the down‐stream signaling axes of MEK/ERK^28^ and/or JNK/c‐Jun are closely associated with the IL‐1α‐stimulated expression of neprilysin in aHDFs. Since up‐regulation of the c‐FOS protein occurs down‐stream of the MEK/ERK^28^ and JNK/c‐Jun axes and in association with c‐Jun, it generates the AP‐1 complex that has a DNA‐binding activity,^29^ we explored whether transfection of a c‐FOS siRNA could abrogate the IL‐1α‐increased expression of neprilysin at the mRNA and/or enzymatic activity levels. In this study, while IL‐1α significantly stimulated the protein level of c‐FOS at 2 h post‐treatment, transfection of the c‐FOS siRNA significantly abrogated the IL‐1α‐increased protein level of c‐FOS, accompanied by a significant abolishment of the enhanced protein and activity levels of neprilysin. The sum of these results strongly suggests that the IL‐1α stimulated expression of the wrinkle‐inducing elastase neprilysin in aHDFs could be mediated via activation of the intracellular signaling axis of ERK/JNK/c‐Jun/c‐Fos/AP‐1, as shown in Figure 7, although to substantiate such a conclusion, a reasonable explanation of the following questions would be required.

First, since c‐FOS expression is mediated via CREB activation and its transcriptional activity,^20^, ^30^ the up‐regulated protein levels of c‐FOS in IL‐1α‐treated aHDFs are thought to be ascribed to the increased phosphorylation (activation) of both the p38/MSK1/CREB and the MEK/ERK/RSK/CREB axes. Thus, it still remains unclear why signaling inhibitors of p38 and MSK1 could not abrogate the IL‐1α‐enhanced neprilysin activity because of their possible inhibitory effects on the IL‐1α‐increased c‐FOS protein due to the abrogated activation of the p38/MSK1/CREB/c‐FOS axis. It is probable that abrogation of the IL‐1α‐increased protein level of c‐FOS by inhibitors of p38 and MSK1 is not fully responsible for attaining the significant abolishment of the IL‐1α‐enhanced activity of neprilysin because the up‐regulated protein levels of c‐FOS mediated via activation of the MEK/ERK/RSK/CREB axis still occur at levels sufficient to stimulate neprilysin activity in the inhibitor‐treated and IL‐1α‐exposed aHDFs.

Second, it still remains unclear as to why the signaling inhibitors of MEK could completely abrogate the IL‐1α‐enhanced neprilysin activity despite the fact that unabrogated activation of the p38/MSK1/CREB/c‐FOS axis still occurs in MEK inhibitor‐treated and IL‐1α‐exposed aHDFs, which may elicit an increased c‐FOS protein at a level sufficient to stimulate neprilysin activity. It is also known that the down‐stream axis of ERK associated with the increased expression of c‐FOS includes both the MSK1/CREB/c‐FOS and the RSK/CREB/c‐FOS axes and the phosphorylation of c‐FOS by activated ERK and RSK,^31^, ^32^, ^33^ as well as, most importantly, the direct ERK/c‐FOS axis,^28^ all of which could be abrogated by the MEK inhibitor. Thus, it is likely that the MEK inhibitor could efficiently inhibit the phosphorylation both of ERK/MSK1/CREB and of ERK/RSK/CREB as well as the phosphorylation and the expression^28^ of c‐FOS to result in attenuating the DNA binding activity of AP‐1, which would lead to the significant down‐regulation of neprilysin expression.

In conclusion, the sum of our findings and speculations strongly suggests that the IL‐1α stimulated expression of the wrinkle‐inducing elastase neprilysin in aHDFs is mediated via activation of the intracellular signaling cascade of ERK/JNK/c‐Jun/c‐Fos/AP‐1. Since many natural materials and chemicals have been proven to elicit inhibitory effects on MEK/ERK/JNK/RSK signaling factors,^34^, ^35^, ^36^, ^37^, ^38^ our discovery of the intracellular signaling cascade associated with the IL‐1α‐stimulated expression of neprilysin should facilitate the development of new effective anti‐wrinkling agents in the future.

## FUNDING INFORMATION

This research received no official external funding.

## CONFLICT OF INTEREST STATEMENT

The authors have received research funds and conference registration fees from TourtVert Co, Ltd., for this work.

## Data Availability

All data were described in the text of our manuscript.
